# Pan-cancer analysis identifies migrasome-related genes as a potential immunotherapeutic target: A bulk omics research and single cell sequencing validation

**DOI:** 10.3389/fimmu.2022.994828

**Published:** 2022-11-03

**Authors:** Yan Qin, Jie Yang, Cao Liang, Jun Liu, Zhixing Deng, Binli Yan, Ying Fu, Yinghua Luo, Xiaozhen Li, Xiaoying Wei, Wei Li

**Affiliations:** ^1^ Department of Health Management, The People’s Hospital of Guangxi Zhuang Autonomous Region and Research center of Health Management, Guangxi Academy of Medical Sciences, Nanning, Guangxi, China; ^2^ Department of Surgical Oncology, Guangxi Medical University Cancer Hospital, Nanning, Guangxi, China

**Keywords:** migrasome, pan-cancer, tumor immunity, tumor microenvironment, immune checkpoint inhibitors, scRNA-seq

## Abstract

**Introduction:**

The migrasome is a newly discovered organelle that resembles extracellular vesicles in structure. However, the function of the migrasome in tumors, particularly in relation to tumor immunity and tumor microenvironment, is unclear.

**Methods:**

Gene expression data, copy number variation raw data, and methylation data of 33 cancer types were downloaded from The Cancer Genome Atlas database. Immunohistochemistry (IHC) based on 114 case of colorectal cancer was used to validate the expression of the migrasome hub-gene. We analyzed the expression, prognosis, genetic variation, and drug sensitivity profiles of migrasome-related genes (MRGs) in pan-cancer datasets. A migrasome score was constructed based on gene set enrichment analysis, and the correlation of migrasomes with the tumor microenvironment was assessed. The CancerSEA was used to perform a single-cell level functional analysis of the migrasome. Additionally, we also analyzed the correlation between migrasomes and tumor mutational burden (TMB), microsatellite instability (MSI), and tumor immune dysfunction and exclusion scores. Single-cell transcriptome sequencing (scRNA-seq) data was used to assess the activation state of migrasomes in the tumor microenvironment.

**Results:**

PIGK expression was significantly up-regulated in 22 of 33 tumors, and high expression of migrasome was estimated to have contributed to poor prognosis. Missense mutations are the most common type of mutation in MRGs. We identified piperlongumine as a potential drug targeting migrasomes. The migrasome score was significantly and positively correlated with the tumor immunity score and the stroma score. In most tumors, the abundance of macrophages in the tumor microenvironment was significantly and positively correlated with the migrasome score. Additionally, the migrasome scores were significantly correlated with the immune checkpoint genes in pan-cancer as well as immune checkpoint therapy-related markers including TMB and MSI. According to scRNA-seq analysis, migrasome differed significantly among cells of the tumor microenvironment. IHC confirmed low expression of ITGA5 and PIGK in colorectal cancer.

**Discussion:**

We performed the first pan-cancer analysis of migrasomes and discovered that they play an important role in tumor development and immune escape. Our study provides new insights into the role of migrasomes in tumor prognosis and immunotherapy.

## Introduction

Extracellular vesicles are key mediators of material exchange and signaling be among tumor cells and mesenchymal cells in the tumor microenvironment (TME), both locally and remotely. Many aspects of tumorigenesis and tumor-related pathology, including immune regulation, vascular permeation, and stromal remodeling, are influenced by extracellular vesicles (1). Extracellular vesicles can be used as disease targets due to the numerous disease processes involved, and they are important for shaping the local immune state before tumor metastasis and during the tumor metastasis process. As a newly discovered organelle with an extracellular vesicle-like structure in recent years (2), migrasome is expected to be used as biomarkers for predicting tumor progression, recurrence, and metastasis. Specific drugs developed to regulate the production and regulation of tumor migrasome could also lead to new research ideas for the treatment of tumors.

Migrasome is a vesicle-like structure that arises from the tips or intersections of contractile filaments produced at the tail of a cell during migration. The interior of the migrasome contains a variable number of small vesicles that appear as pomegranate-like structures under transmission electron microscopy. When the cell migrates, the tail of the cell produces a series of longer tubular structures called contractile filaments. Unlike extracellular vesicles, contractile filaments connect the migrasome to the cell. In the course of exploring the function of the migrasome, we found that after the migrasome forms and stabilizes, it eventually ruptures and releases its contents into the extracellular environment. Migracytosis is the process by which cells release cytoplasmic components into the external environment through the migrasome. There is still uptake by surrounding cells before the migrasome is ruptured. To mediate both processes, contractile filament contraction is required (3). As a result, we speculate that migrasome may mediate long-distance communication between cells as well as the release of intracellular substances.

Migrasome was found in a large number of cells. Currently, the presence of migrasome has been reported in normal rat kidney (NRK) cells, mouse L929 fibroblasts, MGC803 cell, mouse embryonic fibroblasts MEF and NIH3T3 and human immortalized epidermal cells HaCaT (2–4). In a number of high migratory cancer cells, such as human breast cancer cells MDA-MB-231, human colon cancer cells HCT116 and SW480, migrasomes can be produced after induction (3). The formation of migrasome is dependent on cell migration (5). In the development of tumorigenesis, cytokines, chemokines, signaling pathways, and key proteins in the migrasome play important roles. Pancreatic cancer cell-derived migrasome induces a suppressive immune microenvironment and promotes tumor progression (6). As a result, we speculate that migrasome activity may be related to the invasion and migration of cancer cells, as well as tumorigenesis. We observed the structure of a small number of X-dye labeled migrasome in cancer tissue samples from patients with cancer. During tumor metastasis, cancer cells enter the bloodstream and interact with endothelium and endothelial cells, and this interaction has the potential to produce a large number of migrasome, which may have a significant role in regulating the immune microenvironment of the body after entering the bloodstream. This has only been demonstrated in a few cancers (7). Therefore, more *in vivo* models, especially tumor metastasis models, are required to investigate the process and function of the migrasome. Additionally, it was found that pancreatic cancer cells can produce a large number of migrasome *in vivo* during their growth and movement. Tumor cells secrete chemokines such as CXCL5 through the migrasome to promote the infiltration of immunosuppressive cells into the interior of the tumor tissue and suppress the inflammatory response, promoting the development of tumor (8, 9). CXCL5 is highly expressed in hepatocellular and prostate cancers (10, 11), as well as cholangiocarcinoma (12) and esophageal squamous carcinoma (13), promoting tumor growth and metastasis as a key protein involved in the regulatory of cancer immunity enriched in the migrasome. This also suggests that the migrasome could be used as a predictive biomarker for immunotherapeutic efficacy.

In the previous study, TSPAN4 (Tetraspanin4) was reported to be required for migrasome formation (14). Integrins have also been previously reported to play an important biological role in the formation of migrasome, with ITGB1 (integrin β1) and ITGA5 (integrin α5) from the integrin family being enriched in migrasome and suggested as possible specific markers for migrasome detection (4). NDST1 ((bifunctionalheparan sulfate N-deacetylase/N-sulfotransferase 1) is an enzyme responsible for N-sulfation during heparan sulfate (HS) and heparin biosynthesis (15). PIGK encodes a key component of glycosylphosphatidylinositol (GPI) transamidase (16). EOGT (EGF domain-specific O-linked N-acetylglucosaminetransferase) acts as a key participant in glycosylating NOTCH1, and has been described as a poor prognostic factor for pancreatic cancer (17). CPQ (carboxypeptidase Q) encodes a metallopeptidase belonging to the M28 peptidase family, which is thought to play an important role in the hydrolysis of circulating peptides in human plasma (18). NDST1, PIGK, EOGT and CPQ (carboxypeptidase Q) were enriched in the migrasome and were described as marker proteins of migrasome in the previous research (19).

The extracellular vesicle function has been applied to cancer therapy, and the inhibition of extracellular vesicle-mediated processes has a very typical relevance in cancer therapy. However, the published papers on the pan-cancer studies of migrasome are not comprehensive. Considering the role of migrasome in tumorigenesis and metastasis, we not only evaluated the expression of the migrasome and its relation to the cancer patients’ prognosis but also their role in methylation level analysis, tumor purity, and drug sensitivity analysis. The findings of this study will contribute to further studies on the migrasome and the treatment of patients with tumors.

## Methods

### Single-cell transcriptome sequencing (scRNA-Seq) and data analysis

The single-cell sequencing datasets was derived from two kidney renal clear cell carcinoma (KIRC) samples (GSE152938) from patients undergoing radical nephrectomy. The patients were not receiving any anti-tumor treatment therapy prior to sampling, including chemotherapy, radiotherapy, immunotherapy and Chinese medicine. The single-cell sequencing and analysis methods was performed as previously described. The Hiseq X10 (Illumina, San Diego, CA) was used to sequence all the samples using standard parameters. CellRanger (version 3.0.2) was used to convert preliminary sequencing files (.bcl) to FASTQ files. R (version 3.5.2) and Seurat R package (version 3.1.1) were used for QC and secondary analysis. Integration, de-batching, and standardization were performed before comparison. In addition, we downloaded publicly available single-cell sequencing datasets from the GEO database (GSE188711 and GSE163558). GSE188711 was from three left-sided and three right-sided CRC patients included 27,927 single human CRC cells (20). GSE163558 was from three STAD patients (21).

### Paraffin-embedded tissue collection

Paired cancers and paracancerous tissues were derived from 114 colorectal cancer patients a from the Affiliated Cancer Hospital of Guangxi Medical University, respectively. All patients were diagnosed with colorectal cancer and had not received chemotherapy or radiotherapy before tissue collection. Written informed consent was acquired from all patients. The study was approved by the Ethics and Anthropology Committee of the Affiliated Cancer Hospital of Guangxi Medical University. All experiments and methods were performed in accordance with relevant guidelines and regulations.

### Immunohistochemical staining

All cancer specimens were immersed in formalin. Before staining, tissues were cut to 5 μm thickness and placed on glass slides. Endogenous peroxidase activity was inhibited and blocked by de-paraffinizing, rehydrating, and using 5% bovine serum albumin at 37°C for 30 min. The treated sections were incubated with anti-ITGA5 and anti-PIGK at 4°C overnight, respectively. After that, incubation with secondary anti-peroxidation sunflower at 37°C for 30 minutes is required. After washing three times again with PBS, the sections were developed in diaminobenzidine and microscopic images were made by light microscopy.

### Data collection

We downloaded The Cancer Genome Atlas (TCGA) and Genotype-Tissue Expression (GTEx) RNA expression profiles and clinical data from the UCSC XENA database. Copy number variation (CNV) data and methylation data were collected from The Cancer Genome Atlas (TCGA). Gene expression data of normal tissues were obtained from GTEx.

### CNV analysis

We analyzed and processed CNV raw data from 33 cancer types (N=11,495) using GISTICS2 (22). We calculated the percentage of CNV subtypes using GISTIC-processed CNV data. Only genes with a CNV >5% were considered significant. This approach was adopted by Schlattl et al (23). The TCGA barcodes of the samples were used to merged mRNA expression and CNV data. Pearson product-moment correlation coefficient and t-distribution were used to find associations between paired mRNA expression and CNV percentage.

### Differential analysis of DNA methylation

We used the TCGA database to download and analyze methylation data from paired tumor and normal samples from 14 patients with cancer (N = 10,129). CpGs with missing values >10% were removed. Then, using paired t-tests, differentially methylated CpGs between tumor and adjacent normal tissue were identified. The false discovery rate (FDR) method was used to adjust the P-values. CpGs with FDRs <0.05 and absolute beta differences >0.2 were considered differentially methylated.

### Prognostic analysis

The influence of genes on the prognosis of different cancers can be assessed using univariate Cox regression analysis and Kaplan-Meier mapper4. We used Kaplan–Meier and univariate Cox regression analyses to analyze the correlation between overall survival (OS), disease-specific survival (DSS), and progression-free interval (PFI) of MRGs in TCGA. p < 0.05 was considered statistically significant.

### Immunomodulatory analysis

We used TCGA data to analyze the correlation between MRGs and immune regulation. A heat map depicts the correlations between MRG expression and immune-activating genes, immune-suppressing genes, chemokines, and chemokine receptors. p < 0.05 was considered statistically significant.

### Tumor immune microenvironment analysis

We used the RAID algorithm to assess the expression of MRGs in the TME. The immune score, stromal score, and estimate score all reflect the degree of immune cell infiltration.

### Drug sensitivity analysis

Following the method used by Rees et al. (24), 481 small molecules were collected from the Cancer Therapy Response Portal (CTRP). We performed drug sensitivity analysis of MRGs among 30 drugs.

### Migrasome score evaluation

The migrasome score was calculated based on the single-sample gene-set enrichment analysis (ssGSEA) using the MRG set. MRG collection is from published papers (19). R software gene set variation analysis package was used to perform the ssGSEA, which calculated the enrichment score of a gene set in a given sample (25).

### Pathway exploration for migrasome at the single-cell level

We searched the CancerSEA database to further understand the functions and pathways of migrasome in pan-cancer. The CancerSEA database (http://biocc.hrbmu.edu.cn/CancerSEA/) (26) provides information on the different functional status of specific genes in different cancer types at the single-cell level, allowing researchers to bypass tumor heterogeneity. Based on the CancerSEA database, we performed correlations between migrasome and functional status in pan-cancer.

### Immunotherapy response marker analysis

We analyzed MSI and TMB using the expression of MRGs in TCGA and GTEx. Jiang et al. designed Tumor Immune Dysfunction and Rejection Score (27), a novel computational architecture for two tumor immune escape mechanisms. The results could also be used to replace a single biomarker in predicting the effectiveness of immune checkpoint inhibition therapy. We used the Tumor Immune Dysfunction and Exclusion (TIDE) algorithm to predict potential ICB responses. TIDE uses a group of gene expression characteristics to estimate tumor immunization, tumor wetting cytotoxic T lymphocytes (CTL) dysfunction and immunosuppressive factors exclude two different mechanisms of CTL. Patients with high TIDE scores showed higher tumor immunity escape risks, so the reaction rate for ICB treatment is low.

### Statistical analysis

We estimated the prognostic significance of the indicators using Kaplan–Meier survival curves. The log-rank test yielded P values <0.05, which were considered statistically significant.

## Results

### Differential expression of migrasome-related genes and the prognostic impact

**Figure 1:**
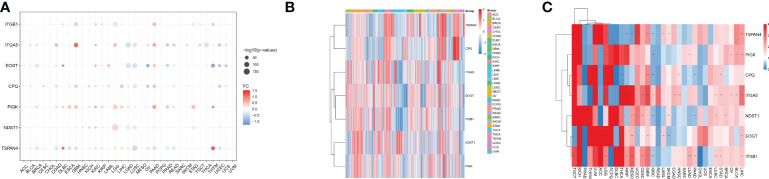
Differential expression of MRGs in pan-cancer correlates with prognosis. **(A)** Differential expression of MRGs in solid tumors in The Cancer Genome Atlas cohort pan-cancer data. Expression analysis was based on the comparison of TCGA tumors with TCGA paracancerous and GTEx normal. **(B)** Expression of the seven MRGs in different cancers. The -2 to 2 values of the color bar in the figure represent the gene expression values after homogenization. **(C)** The relationship between MRGs and cancer prognosis in different cancers. the 0-4 values of the color bar in the figure represent the hazard ratio. **P < 0.01, and ***P < 0.001. FC, Fold change.

We analyzed seven migrasome genes, namely ITGB1, ITGA5, EOGT, CPQ, PIGK, NDST1, and TSPAN4. Based on the TCGA database, we compared the expression of MRGs in 33 tumors (**Supplementary Table 1**). Among the 33 tumors, PIGK expression was significantly up-regulated in 22 tumors, and there was no tumor with significantly down-regulated expression. ITGB1 expression was significantly increased in 15 of 33 tumors, while significantly decreased in 4 of 33 tumors. NDST1 was significantly upregulated in 9 of 33 tumors, while significantly decreased in 13 of 33 tumors.(**Figure 1A**; P < 0.05, **Supplementary Table 2**). We evaluated the expression model of MRGs in different tumors and found that TSPAN4 was highly expressed in KIRC, KIRP, MESO, READ, and UVM; CPQ was highly expressed in THCA (**Figure 1B**).

We further analyzed the relationship between MRGs and cancer prognosis. Among the 33 tumors, TSPAN4 was a prognostic risk factor in six tumors and a protective factor in 3. ITGA5 was a prognostic risk factor in 14 tumors and there were no tumors where ITGA5 was a protective factor (**Figure 1C**; P < 0.05).

### Analysis of mutations in migrasome-related genes

**Figure 2:**
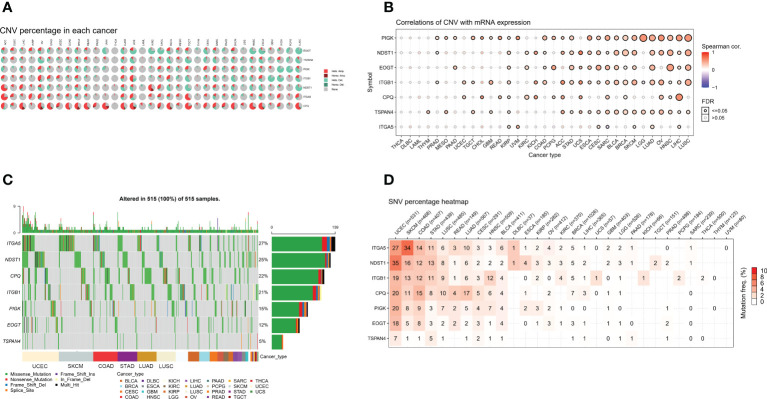
MRGs are present in a large number of genetic variants in pan-cancer. **(A)** Copy number variation (CNV) in the MRGs. CNV pie charts show the heterozygous/homozygous CNV for each gene in each cancer for this combination. **(B)** Correlation of the migrasome mRNA expression with CNV. The statistical signal is represented by the size of the dots, where the larger the dot, the greater the statistical signal. **(C)** The SNV oncoplot shows the distribution of mutations, MRGs, and migrasome SNV types. Sidebar and top bar show the amount of mutations in each gene. **(D)** SNV frequencies of genes in cancer. The higher the mutation frequency, the darker the color. The percentage of samples with the respective mutated gene for a given cancer is indicated by numbers.

We analyzed CNV data from the TCGA database to detect migratory chromosomal variants. The results showed that ITGA5, NDST1, CPQ, ITGB1, PIGK, EOGT, and TSPAN4 had amplifications or deletions in most cancers. We found that EOGT had heterozygous deletion up to 80% in CHOL; TSPAN4 had high heterozygous deletion in OV; PIGK had high heterozygous deletions in KICH and PCPG; ITGB1 had high heterozygous deletions in GBM and KICH, but nearly 50% heterozygous amplification in UCS; NDST1 had high heterozygous deletions in TGCT and LUSC, but also very high heterozygous amplification in ACC; ITGA5 had high heterozygous amplifications in ACC and TGCT; CPQ had heterozygous amplification up to 50% in STAD, COAD, READ, UVM, LUAD, UCS, BLCA, TGCT, HNSC, ESCA, and LUSC (**Figure 2A**). We analyzed the relationship between MRGs mRNA expression and CNV to further investigate the correlation between migrasome genes and CNV. Among 33 tumors, PIGK expression was significantly positively correlated with CNV in 25 tumors, NDST1 expression was significantly positively correlated with CNV in 19 tumors, EOGT expression was significantly positively correlated with CNV in 17 tumors, ITGB1 expression was significantly positively correlated with CNV in 21 tumors, and negatively correlated with LGG (**Figure 2B** and **Supplementary Table 3**).

We used data from migrasome-associated SNPs to detect mutations and their frequency in various cancers. The graphs showed a significant increase in SNV frequencies for UCEC, SKCM, COAD, and STAD. The SNV frequencies of ITGA5, NDST1, CPQ, ITGB1, PIGK, EOGT, and TSPAN4 were 27%, 25%, 22%, 21%, 15%, 12%, and 5%, respectively (**Figure 2C**). ITGA5, NDST1, ITGB1, CPQ, PIGK, EOGT, and TSPAN4 had a percentage heatmap of 27%, 35%, 19%, 20%, 20%, 18%, and 7% at UCEC; 34%, 16%, 13%, 11%, 8%, 5%, and 1% at SKCM; 14%, 12%, 12%, 15%, and 9% at COAD, 8%, and 1% at STAD; and 11%, 13%, 11%, 8%, 3%, 3%, and 5% at STAD, respectively (**Figure 2D**). These findings suggested that mutations in the migrasome were associated with tumorigenesis.

### Methylation analysis of migrasome-related genes

**Figure 3:**
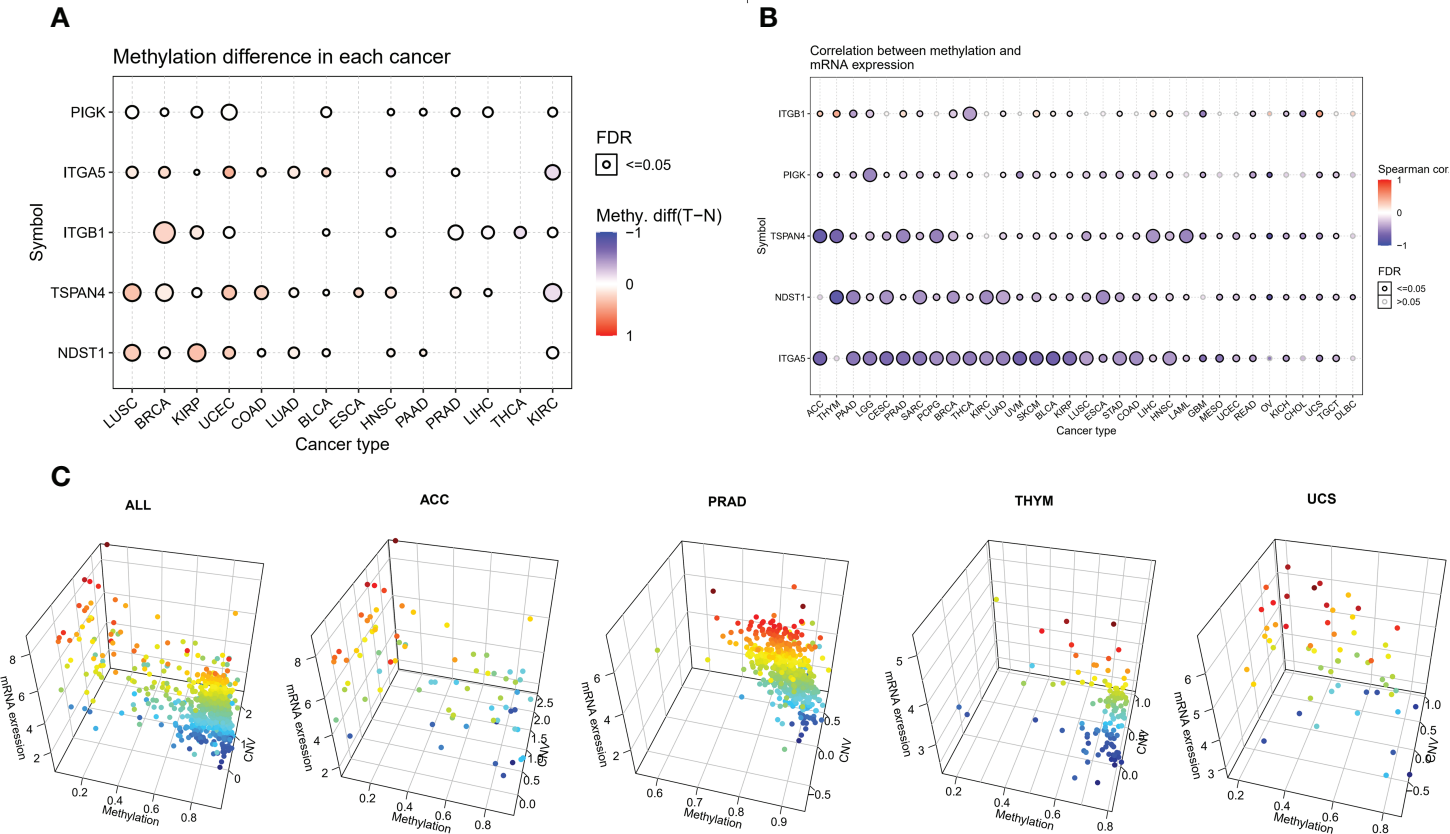
Aberrant methylation of MRGs in pan-cancer. **(A)** The methylation levels of MRGs between tumor and normal samples for each cancer. Blue dots indicate low tumor methylation levels and red dots indicate high tumor methylation levels. **(B)** Correlation of methylation with the mRNA gene expression. Blue dots indicate a negative correlation, while red dots indicate a positive correlation, and the deeper the color, the higher the correlation. **(C)** Correlation analysis of ITGB1 expression with methylation and CNV.

We analyzed the methylation of MRGs to determine methylation levels. Among the 14 tumors in which the methylation level of cancer and adjacent tissues could be obtained, the methylation level of PIGK was significantly increased in 10 tumors, the methylation level of ITGA5 was significantly increased in 8 tumors and significantly decreased in 2 tumors, and the methylation level of ITGB1 was significantly increased in 6 tumors and significantly decreased in 3 tumors (**Figure 3A** and **Supplementary Table 4**). To further explore the relationship between MRGs and methylation, we analyzed the association between migrasome mRNA expression and methylation using samples from the TCGA database. The results showed that the expression of migrasomal genes ITGB1, ITGA5, NDST1, TSPAN4, and PIGK were negatively associated with 22 tumors, including AAD, LGG, CESC, PRAD, SARC, PCPG, BRCA, THCA, LUAD, UVM, SKCM, BLCA, KIRP, LUSC, ESCA STAD, COAD, LIHC, HNSC, READ, UCS, and TGCT, but ITGB1 was positively associated with methylation in ACC, THYM, PRAD, SKCM, BLCA, LIHC, HNSC, and UCS (**Figure 3B**). We further analyzed the mechanisms behind the positive correlation between ITGB1 expression and methylation. We extracted ITGB1 mRNA expression values, methylation values and CNV values in ACC, THYM, PRAD and UCS and performed correlation analysis. We found that ITGB1 expression was negatively correlated with methylation and positively correlated with CNV in these tumors. This was also observed in each of the tumors (**Figure 3C**). We speculate that the positive correlation between ITGB1 expression and methylation may be due to a compensatory CNV mechanism.

### Drug sensitivity analysis of migrasome-related genes

**Figure 4:**
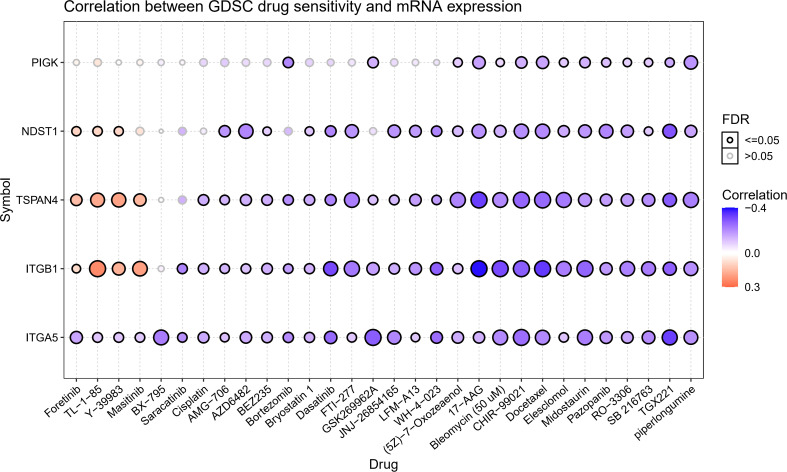
Expression of MRGs correlates with drug sensitivity. A positive correlation indicates that a highly expressed gene is drug-resistant and vice versa.

We gathered data in CTRP to better understand the role of migrasome in cancer treatment. The results showed that the drugs 5Z-7-Oxozeaenol, 17-AAG, Bleomycin (50 uM), CHIR-99021, Docetaxel, Elesclomol Midostaurin, Pazopanib, RO-3306, SB 216763, TGX221, and piperlongumine were associated with ITGB1, ITGA5, NDST1, TSPAN4, and PIGK. Resistance to the drugs Foretinib, TL-1-85, and Y-39983 was associated with ITGB1, NDST1, and TSPAN4, and resistance to the drug Masitinib was associated with ITGB1 and TSPAN4 (**Figure 4**). These results suggested that the migrasome may have an impact on the therapeutic outcomes of cancer.

### Differential expression and correlation with the stage of migrasome scores

**Figure 5:**
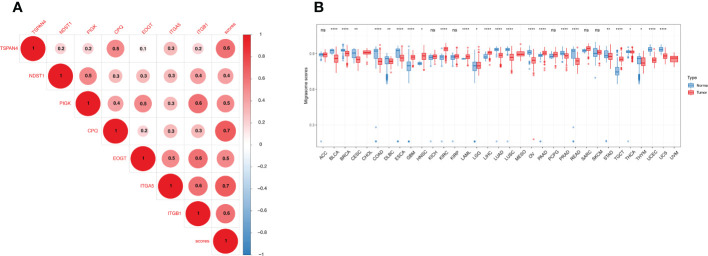
Expression of the migrasome scores. **(A)** Association of the MRGs. Higher values represent stronger correlations. **(B)** Migrasome expression in tumor and normal tissues of The Cancer Genome Atlas and The Genotype-Tissue Expression cohorts. Data are represented as mean ± standard deviation. *P < 0.05, **P < 0.01, and ****P < 0.0001, ns, no significant.

We constructed a migrasome score based on the ssGSEA algorithm to comprehensively assess the migrasome status. We explored the association between scores were and MRGs, and the results showed that migrasome scores were significantly and positively correlated with MRGs (P < 0.05; R > 0.5). Additionally, we observed significant strong correlations between MRGs, suggesting a close association between MRGs (**Figure 5A**).

We analyzed the samples in TCGA and GETx to see if migrasome expression was present in tumor tissues. The results showed that migrasome score increased significantly in 8 of 33 tumors and decreased significantly in 16 of 33 tumors. (**Figure 5B**; P < 0.05). We further analyzed the relationship between the migrasome score and the tumor stage. The results suggested that migrasome score increased significantly only in the advanced stage of BLCA (**Supplementary Figure 1**; P < 0.05, and **Supplementary Table 5**).

### Prognostic significance of migrasome scores in cancer

**Figure 6:**
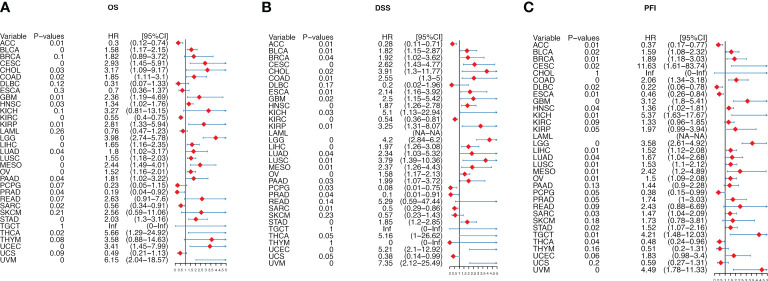
Migrasome scores are strongly correlated with patient prognosis. **(A)** Forest plot shows univariate Cox regression findings of migrasome on overall survival in The Cancer Genome Atlas (TCGA) pan-cancer. **(B)** The Forest plot shows the univariate Cox regression findings of migrasome on disease-specific survival in TCGA pan-cancer. **(C)** The Forest plot shows the univariate Cox regression findings of migrasome on the progression-free interval in TCGA pan-cancer. The red color represents significant outcomes.

We further analyzed the prognostic significance of migrasome in patients with cancer. We analyzed the prognostic relationship of migrasome using univariate COX regression analysis. The OS results showed that the migrasome score was a protective factor in 3 of 33 tumors (ACC, KIRC and PRAD) and a risk factor in 18 tumors (**Figure 6A**; P < 0.05). The DSS results showed that the migrasome was a protective factor for ACC, KIRC, PCPG, PRAD, and SARC, and a risk factor in 20 of 33 tumors, including BLCA, BRCA, CESC, CHOL, COAD, ESCA, GBM, HNSC, KICH, KIRP, LGG, LIHC, LUAD, LUSC, MESO, OV, PAAD, STAD, UCEC, and UVM (**Figure 6B**; P < 0.05). The PFI results showed that the migrasome was a protective factor for ACC, DLBC, ESCA, and THCA, and a risk factor in 17 of 33 tumors, including BLCA, BRCA, CESC, COAD, GBM, HNSC, KICH, LGG, LIHC, LUAD, LUSC, MESO, OV, SARC, STAD, TGCT, and UVM (**Figure 6C**; P < 0.05, **Supplementary Table 5**). These results suggested that a high expression level of migrasome might lead to a poor prognosis. Using Kaplan–Meier analysis, we further analyzed the correlation between migrasome and OS, DSS, and PFI in TCGA. We found that migrasome was negatively correlated with BLCA, CESC, COAD, GBM, HNSC, LGG, LUAD, LIHC, LUSC, MESO, OV, STAD, and UVM in OS, DSS, and PFI (**Supplementary Figures 2**–**4**; P < 0.05). These results suggested that high expression of migrasome in these 13 cancers indicated poor prognosis.

### Relationship between migrasome scores and tumor immune microenvironment

**Figure 7:**
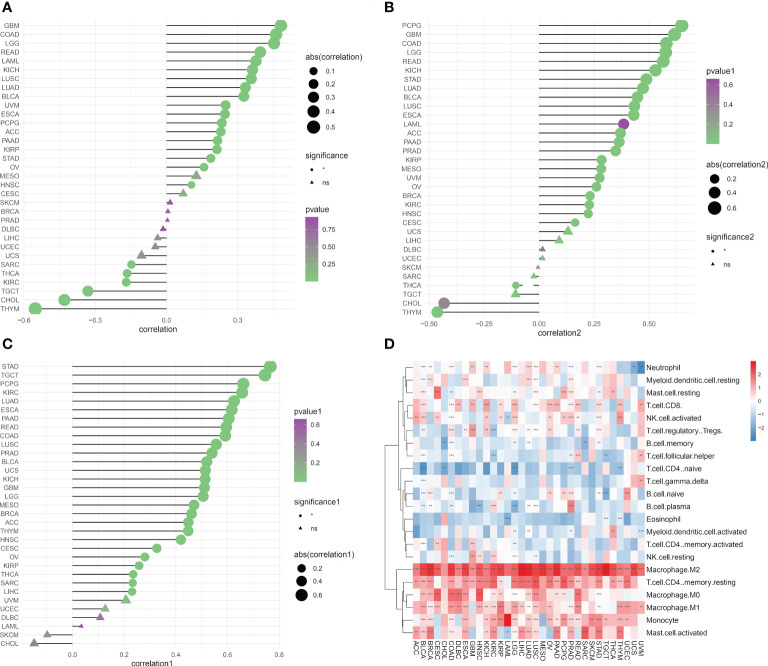
Migrasome scores are closely related to immune cell infiltration. **(A)** Analysis of tumor ImmuneScore. *P < 0.05, **P < 0.01, ***P < 0.001, and NS, no significant. **(B)** Analysis of tumor MicroenvironmentScore. *P < 0.05, **P < 0.01, ***P < 0.001, and NS, no significant. **(C)** Analysis of tumor StromaScore. *P < 0.05, **P < 0.01, ***P < 0.001, and NS, no significant. **(D)** A heat map depicting the correlation between migrasome expression in cancer and tumor immune infiltration. **P < 0.01, ***P < 0.001.

We determined the abundance of migrasome in the tumor microenvironment using the TIMER database. The results showed that in ImmuneScore, StromaScore, and MicroenvironmentScore, migrasomes were positively correlated with HNSC, OV, STAD, KIRP, PAAD, ACC, PCPG, ESCA, UVM, BLCA, LUAD, LUSC, KICH, READ, LGG, and COAD (**Figures 7A–C**; P < 0.05). We further evaluated the correlation between migrasome score and tumor immune infiltration. The results showed that in most tumors, migrasome was positively correlated with Macrophage M2 and T cell CD4 memory resting, but negatively correlated with T cell CD4 naive (**Figure 7D**; P < 0.05).

### Correlation of migrasome scores with immunotherapy response markers

**Figure 8:**
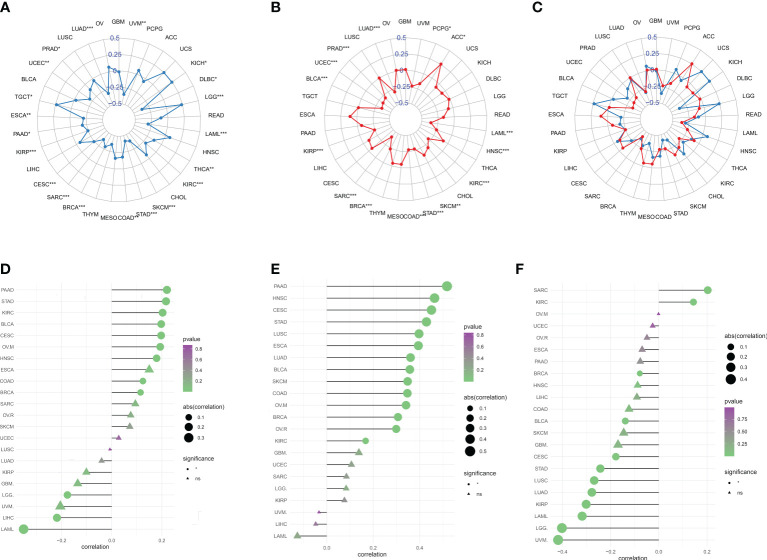
Migrasome scores are closely related to microsatellite instability (MSI) and tumor mutational burden (TMB). **(A)** Correlation between migrasome scores and TMB in human cancers. *P < 0.05, **P < 0.01, ***P < 0.001. **(B)** Correlation between migrasome scores and MSI in human cancers. The Spearman’s correlation coefficient and p-value are shown in the radar plot. *P < 0.05, **P < 0.01, ***P < 0.001. **(C)** Correlation of migrasome scores with TMB and MSI. The blue line represents TMB and the red line represents MSI. **(D)** Correlation between migrasome scores and tumor immune dysfunction and exclusion (TIDE). The higher the TIDE scores, the higher the chance of tumor immune escape. **(E)** Correlation between migrasome scores and TIDE in the high TIDE group. **(F)** Correlation between migrasome scores and TIDE in the low TIDE group. ns, no significant.

Tumor immune escape can be monitored to predict the survival of patients with cancer who are treated with drugs. This suggested that tumor immune escape could be facilitated by migrasome expression. We found that migrasome was negatively correlated with UVM, DLBC, THCA, KIRC, SKCM, STAD, COAD, BRCA, SARC, CESC, KIRP, PAAD, ESCA, UCEC, PRAD, LUAD, and LAML in TMB. However, it was positively correlated with KICH, LGG, and TGCT in TMB (**Figure 8A**; P < 0.05). Migrasome was negatively correlated with PCPG, LAML, HNSC, KIRC, SKCM, STAD, COAD, BRCA, SARC, KIRP, BLCA, UCEC, PRAD, and LUAD in MSI, but positively correlated with ACC in MSI (**Figure 8B**; P < 0.05). Using the TIDE score, we further explored the correlation between the migrasome and markers of immunotherapeutic response. Patients with a high TIDE score had a higher chance of tumor immune escape and poorer drug treatment outcomes. We found that the expression of migrasome was positively correlated with BRCA, COAD, HNSC, OVM, CESC, BLCA, KIRC, STAD, and PAAD (9 of 33), but negatively correlated with LAML, LIHC, and LGG (**Figure 8C** and **Supplementary Figure 5**; P < 0.05). We further conducted subgroup analyses on samples with different TIDE scores. Patients were divided into a high TIDE group versus a low TIDE group according to a median TIDE of 0.004. We calculated the correlation between TIDE and migrasome scores in the different subgroups separately. Interestingly, we found that the migrasome score was positively correlated with the TIDE score in most of the tumors in the high TIDE group and negatively correlated with the TIDE score in most of the tumors in the low TIDE group (**Figures 8D–F**). This result suggests that there is an interaction between the migrasome score and the TIDE score, and that the combination of the two may be more accurate in predicting the effect of immunotherapy.

### Correlation of migrasome scores with immune-related genes

**Figure 9:**
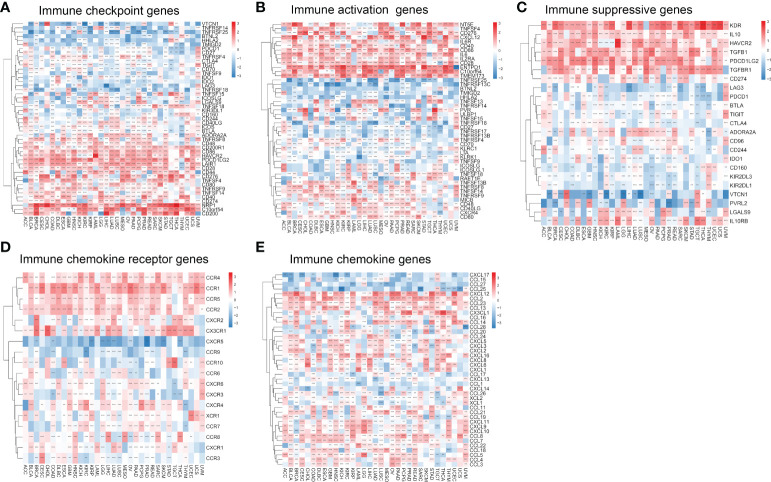
Migrasome scores are closely related to immune-related genes. **(A)** A heat map shows the relationship between immune checkpoint genes and migrasome in different cancers. **(B)** Correlation between immune activation genes and the migrasome in pan-cancer. **(C)** Correlation between immune-suppressive genes and the migrasome in pan-cancer. **(D)** Correlation between chemokine receptor and the migrasome in pan-cancer. **(E)** Correlation between chemokine and the migrasome in pan-cancer. **P < 0.01, and ***P < 0.001.

We analyzed the relationship between migrasome and immune checkpoints. In LAML, the migrasome scores in the monitored immune checkpoints were positively correlated with C10orf54, TNFRSF9, CD44, TNFRSF8, CD48, CD200R1, CD86, HAVCR2, TNFSF15, and LGALS9 (**Figure 9A**; P < 0.05). This suggested that the migrasome could aid tumor immune escape. In pan-cancer, we investigated the relationship between 46 immune activation genes and migrasome. The results showed that CD40, CD86, IL6, IL2RA, CD28, ENTPD1, C10orf54, TMEM173, TNFSF13B, TNFRSF8, TNFSF14, TNFRSF9, MICB, CD48, CD40LG, CXCR4, and CD80 were all positively correlated with the migrasome in most tumors. However, there was a negative correlation between TNFRSF25 and TNFRSF13C (**Figure 9B**; P < 0.05). We further investigated the relationship between the migrasome and immunosuppressive genes. The results showed that in most tumors, the migrasome was positively correlated with KDR, IL10, HAVCR2, TGFB1, PDCD1LG2, and TGFBR1 (**Figure 9C**; P < 0.05). We further investigated the relationship between migrasome and chemokine receptors as well as chemokines. The results showed that in most tumors, the migrasome was positively correlated with chemokine receptors CCR4, CCR1, CCR5, CCR2, CXCR2, and CX3CR1, and was positively correlated with chemokines CXCL12, CCL2, CCL23, CCL13, CXCL16, CCL8, CCL7, CCL22, CCL18, CCL5, and CCL4 (**Figures 9D, E**; P < 0.05). Migrasome may have a strong correlation with immunomodulatory genes.

### Analysis of migrasome scores function at the single-cell level

**Figure 10:**
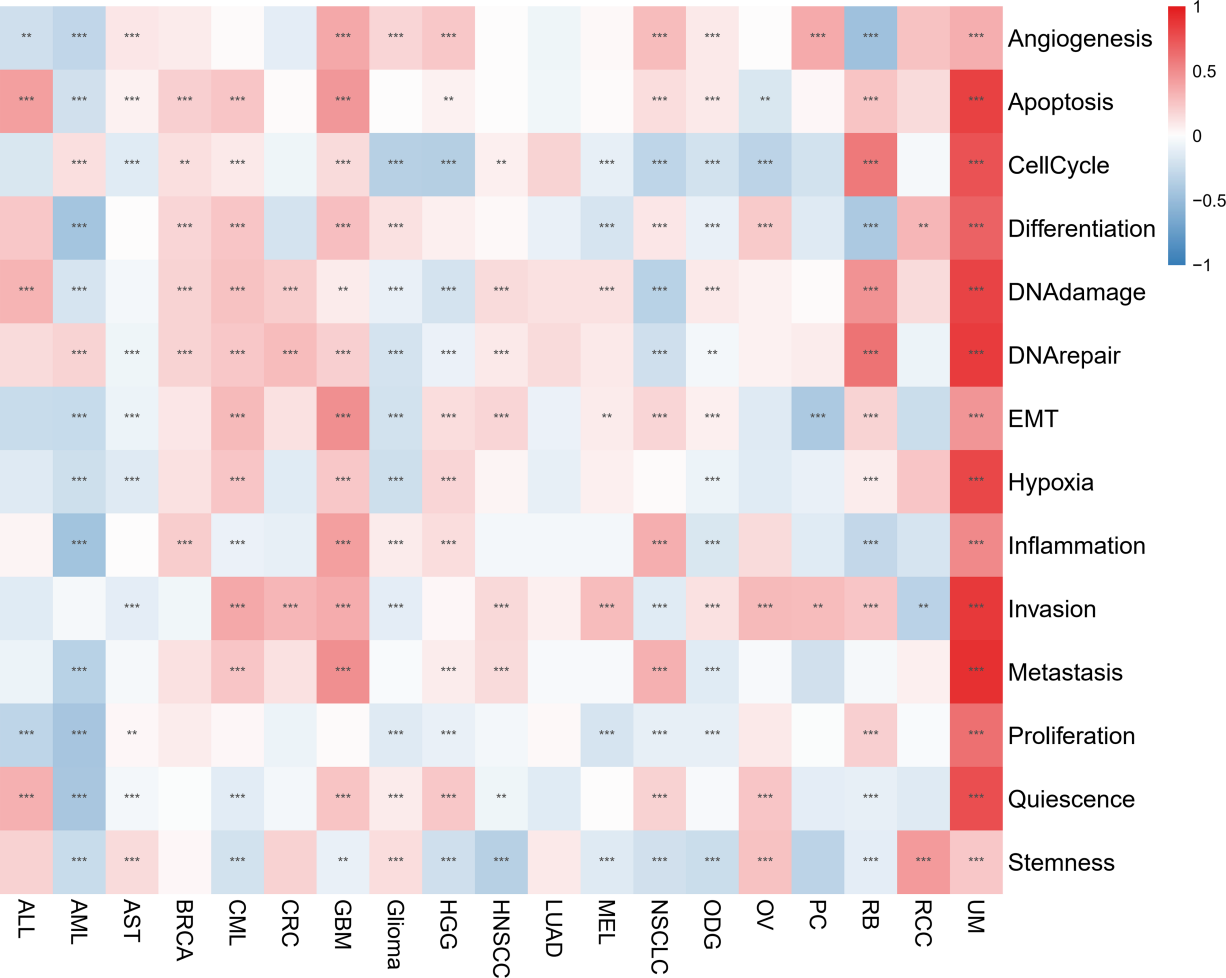
Migrasome scores are closely related to the cellular function of different cancer patients. Red represents positive correlations, while blue represents negative correlations. **P < 0.01, and ***P < 0.001.

The CancerSEA database was used to determine the functional status of the migrasome score in various cancers at the single-cell level. The results showed that the migrasome was positively correlated with angiogenesis, apoptosis, cell cycle, differentiation, DNA damage, DNA repair, EMT, hypoxia, inflammation, invasion, metastasis, proliferation, quiescence, and stemness in UM. Migrasome was negatively correlated with angiogenesis, apoptosis differentiation DNA damage, EMT, hypoxia, inflammation, metastasis, proliferation, quiescence, and stemness in AML. However, no significant functional states associated with migrasome were found in LUAD (**Figure 10**; P < 0.05).

### Single-cell transcriptional analysis of migrasome in the KIRC tumor microenvironment

**Figure 11:**
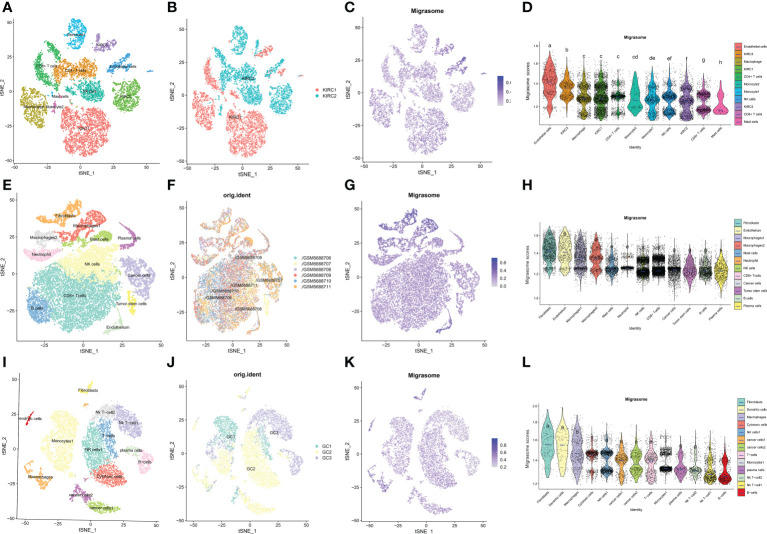
Single cell transcriptome profiles of kidney renal clear cell carcinoma (KIRC), colon carcinoma (COAD) and stomach adenocarcinoma (STAD). **(A)** tSEN plots of KIRC samples shows 11 different cell types. **(B)** tSEN plots of KIRC from two different samples. **(C)** tSEN plots of migrasome fractions represent different cell types. **(D)** Comparison of migrasome scores in different KIRC tumor microenvironment cells. The blue horizontal lines on the graphs indicate the median migrasome scores. The letter at the top indicates whether there is a statistical difference between the two comparisons between cells. A different letter indicates that the difference is statistically significant. **(E)** tSEN plot of COAD samples shows 12 different cell types. **(F)** tSEN plots of COAD for six different samples. **(G)** tSEN plots represent the migrasome fraction of different cell types. **(H)** Comparison of migrasome scores in different COAD tumor microenvironment cells. The blue horizontal lines on the graphs indicate the median migrasome scores. The letter at the top indicates whether there is a statistical difference between the two comparisons between cells. A different letter indicates that the difference is statistically significant. **(I)** tSEN plot of STAD samples shows 13 different cell types. **(J)** tSEN plots of STAD for three different samples. **(K)** tSEN plots represent the migrasome fractions of different cell types. **(L)** Comparison of migrasome scores in different STAD tumor microenvironment cells. The blue horizontal lines on the graphs indicate the median migrasome scores. The letter at the top indicates whether there is a statistical difference between the two comparisons between cells. A different letter indicates that the difference is statistically significant.

ScRNA-seq was performed on two in-house KIRC samples. After QC using Seurat, 13124 high-quality single-cell transcriptome information was used for subsequent analysis. Cell clustering analysis based on the tSNE algorithm showed that the above cells could be classified into 11 clusters, namely KIRC1, KIRC2, KIRC3, monocyte1, monocyte2, macrophage, mast cells, endothelial cells, NK cells, CD4+ T cells, and CD8+ T cells (**Figure 11A**). Different cell clusters had significantly different expression levels of marker genes (**Supplementary Figure 6**). Additionally, we found that tumor cells from two different KIRC sample sources had the same cluster (KIRC3) and unique clusters (KIRC1 and KIRC2) (**Figure 11B**). The above results suggest that KIRC cell types are heterogeneous. We used ssGSEA to impute migrasome scores for KIRC tumor microenvironment cells and compared the differences in migrasome scores across cell types (**Figure 11C**). Interestingly, we found that the migrasome scores of different cells differed significantly (**Figure 11D** and **Supplementary Table 6**). Endothelial cell migrasome scores were significantly higher than those of any other cells, suggesting that endothelial cells’ migrasomes were significantly activated in the KIRC tumor microenvironment (P < 0.05). Mast cells had the lowest migrasome score. Migrasome scores differed significantly among the different KIRC cell clusters, suggesting that the migrasome may be a characteristic of KIRC cells. These results suggest that migrasome are significantly different in different cells of the KIRC tumor microenvironment and that targeting migrasome could be a breakthrough in regulating the tumor microenvironment. We used publicly available scRNA-seq data from COAD and STAD for further analysis. scRNA-seq based on COAD showed that fibroblasts, endothelial cells and macrophages had the top three abundances of migrasome scores (**Figures 11E–H** and **Supplementary Figure 7**). scRNA-seq based on STAD showed that migrasome scores were in the top three for fibroblasts, dendritic cells and macrophages (**Figures 11I–L** **Supplementary Figure 8**). The results showed some similarity with the scRNA-seq results from KIRC, i.e. both endothelial cells and macrophages had higher migrasome scores. It suggests that the migrasome of endothelial cells and macrophages in the tumor microenvironment may be more active.

### Immunohistochemical validation of ITGA5 and PIGK expression

**Figure 12:**
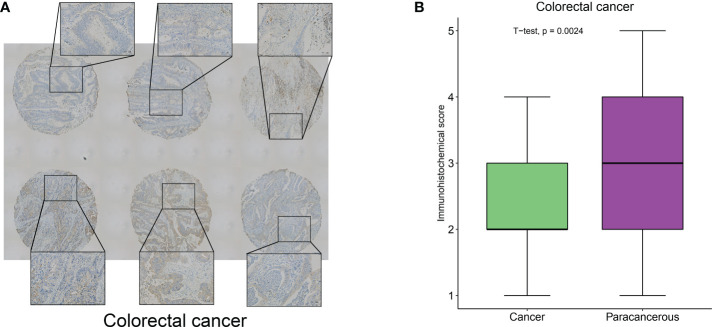
ITGA5 was significantly low expressed in colorectal cancers by Immunohistochemical validation. **(A)** Representative images of immunohistochemical detection of ITGA5 expression in colorectal cancer. **(B)** Statistical results of ITGA5 expression.

To clarify the hub-gene in MRGs, we analyzed the interaction relationships of MRGs using a protein interaction network and the results showed that ITGA5 had the highest number of interactions identified as a hub-gene (Degree=11, **Supplementary Figure 9**). We examined the expression of ITGA5 and PIGK in 114 pairs of colorectal cancers versus normal tissues using immunohistochemistry and showed that ITGA5 was significantly low expressed (**Figures 12A, B**, P=0.0024) and PIGK was significantly high expressed in colorectal cancers (**Supplementary Figure 10A, B**, P=0.023), which was consistent with our analysis.

### Integrated analysis of multiple omics data of migrasome-related genes

**Figure 13:**
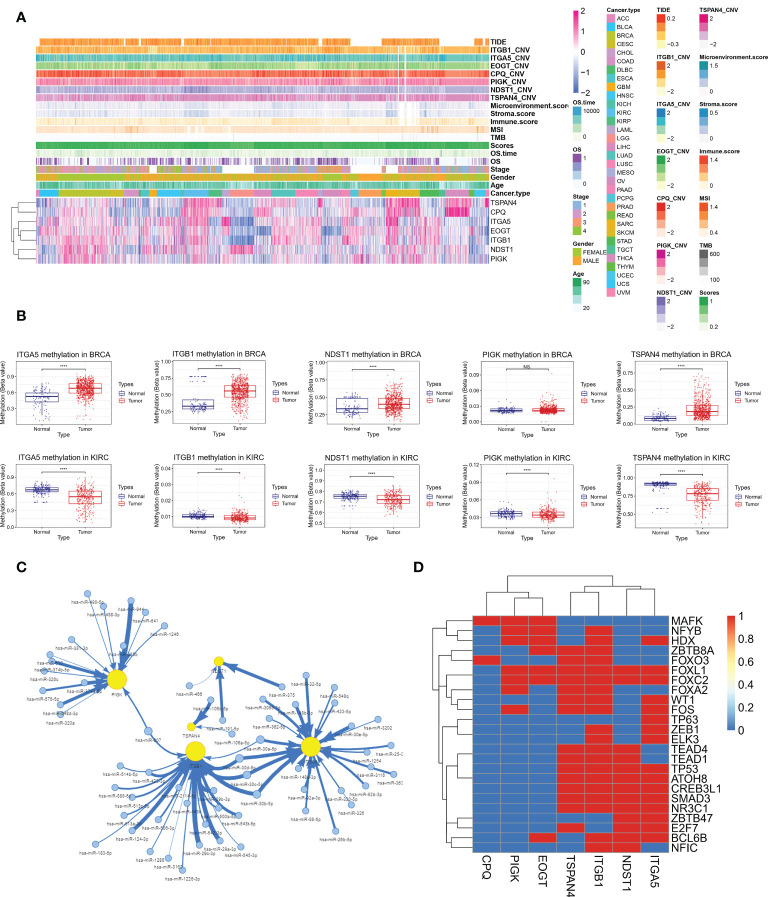
Integrative analysis of multi-omics data of MRGs to find potential co-regulatory mechanisms. **(A)** Correlation of MRGs expression with migrasome score, tumor type, tumor stage, gender, age, overall survival, TMB, MSI, immune microenvironment score, CNV and TIDE score. **(B)** Altered methylation levels of ITGA5, ITGB1, NDST1, PIGK and TSPAN4 in BRCA and KIRC. ****P < 0.0001, NS, no significant. **(C)** The miRNA regulatory network shows miRNA-gene targeting relationships of ITGA5, ITGB1, NDST1, PIGK and TSPAN4 in BRCA and KIRC in BRCA and KIRC. **(D)** Correlation of MRGs and transcription factors.

Correlation of MRGs expression with migrasome scores, tumor type, tumor stage, gender, age, overall survival, TMB, MSI, immune microenvironment score, CNV and TIDE score was shown in **Figure 13A**. We observed a high consistency in the expression pattern of MRGs in some tumors, such as BRCA and KIRC, which suggested that there might be a common regulatory mechanism. We further analyzed the underlying regulatory mechanisms and showed that the methylation levels of ITGA5, ITGB1, NDST1, PIGK and TSPAN4 were significantly upregulated in BRCA and downregulated in KIRC compared to paracancerous tissues (**Figure 13B**). This suggests that aberrant methylation may be involved in the regulation of MRGs expression. miRNA regulatory networks may also coordinate gene expression. We analyzed the miRNA-gene targeting relationships of the above MRGs in BRCA and KIRC. The results showed that among the statistically significant regulatory relationship pairs, has-miR-30a-5p, has-miR-30b-5p, has-miR-30c-5p and has-miR-30d-5p were co-regulators of ITGB1 and ITGA5. hsa-miR-607 was a co-regulator of PIGK and ITGB1(**Figure 13C**). The miRNA regulatory network may partially influence the expression of MGRs. Transcription factors (TF) can also coordinate gene expression. We analyzed the common TFs of MRGs and the results showed that ITGA5, ITGB1, NDST1, TSPAN4 share more TFs, while CPQ, EOGT and PIGK have more intersecting TFs. It was pointed out that FOXL1 and FOXC2 regulated 6 of the 7 MRGs (**Figure 13D**). The above mechanisms were involved in the regulation of MRGs expression in BRCA and KIRC.

## Discussion

Migrasome, which is large vesicles that grow at the tips or intersections of contractile fibers behind migrating cells, has recently been discovered and considered a new organelle (3). The migrasome plays an important role in cell-cell communication and cellular homeostasis. Additionally, signaling molecules, such as chemokines, cytokines, and growth factors are abundant in the migrasome and can be released (2). Therefore, the migrasome could play an important role in tumor metastasis and tumor immune escape. However, no pan-cancer analysis for the role of the migrasome in different cancers has been done, and our study fills this gap.

First, we analyzed the altered expression and prognosis of migrasome and multiple MRGs and found that their abnormal expression was correlated with clinical prognosis. It showed that the expression of these MRGs was differentially upregulated in multiple tumor types compared to paraneoplastic tissues, suggesting that migrasome may play a biological role in the development or progression of multiple tumors. Human breast cancer cell (MDA-MB-231), human colon cancer cell (HCT116), human adenocarcinoma cell (SW480), human gastric carcinoma cell (MGC803), and human ovarian adenocarcinoma cell have all been found to contain migrasome (3, 28). We further analyzed the relationship between multiple genes and multiple tumor prognosis and found that high expression of multiple MRGs was associated with a poorer prognosis. Furthermore, survival analysis showed that overexpression of the migrasome was associated with a poorer OS and DSS, and that expression of the migrasome and its associated genes increased as the cancer stage progressed in most tumor types. These findings suggest that high migrasome expression is associated with poorer prognosis in patients with cancer, possibly contributing to lower patient survival rates. Thus, abnormal migrasome expression may serve as one of the assessment indicators for predicting human pan-cancer.

Alterations in DNA methylation have been noticed in various cancers and are considered to play a part in carcinogenesis (29). We further analyzed the methylation levels of several MRGs to gain a deeper understanding of the mechanism of action of MRGs on multiple tumorigeneses. We found that MRGs were commonly differentially methylated in a variety of tumors, and MRGs were differentially hypermethylated in a variety of tumor types. It could provide a basis for subsequent in-depth research into the methylation of MRGs in the future. Furthermore, by tracing genetic differences in these multiple regulatory factors, we identified that missense mutations were the predominant mutation type in SNV, with ITGA5 having the highest mutation frequency in multiple tumors. It has been reported that ITGA5 mutations are related to the occurrence and development of various tumors, and overexpression of ITGA5 is connected with poor prognosis in a variety of gastrointestinal tumors, while in laryngeal squamous cell carcinoma, high ITGA5 expression is an independent adverse prognostic factor (30, 31). However, our study found that ITGA5 was significantly under-expressed in COAD and we obtained similar results using IHC for validation. Prognostic analysis suggested that ITGA5 was a risk factor for overall survival in COAD. This phenomenon is contrary to conventional knowledge. We speculate that elevated ITGA5 expression may be feedback from increased tumor malignancy, involving other mechanisms regulating ITGA5 expression. Similar phenomena have been reported in KIRC, such as CHAC1 expression was lower in cancer tissues than in normal samples, but was strongly associated with TNM stage and was a prognostic risk factor (32). The role of ITGA5 in COAD, especially in poorly differentiated tumors, needs to be further investigated. Therefore, we hypothesize that these changes in MRGs may cause gene dysfunction and involved in tumorigenesis and development.

Genomic mutations affect the response to clinical therapy and can be potential biomarkers for cancer drug screening. We analyzed the correlation between the expression of related genes and drugs to better understand the effect of MRGs in clinical treatment. The results showed that the expression levels of migrasome-related regulatory genes were correlated with drug sensitivity. Piperlongumine (PL) is a biologically active alkaloid that has been described as an anticancer compound that modulates apoptosis. PL has been reported to exhibit cytotoxic activity against a variety of cancer cells and to inhibit tumor progression through multiple mechanisms, including reactive oxygen species accumulation (33), nuclear factor-κB inhibition (34), ERK activation (35), and telomerase reverse transcriptase activity inhibition (36). Therefore, we believe that targeting MRGs could be a potential tumor treatment approach. However, mutations or variant expressions of MRGs can affect the therapeutic effect of drugs in clinical settings, and this may alter the actual effect of the drug. Thus, the further investigation of each drug’s potential impact mechanisms on migrasome associated gene expression and cancer progression is needed.

Tumor immunotherapy has paved a new pathway for cancer treatment, but with only a limited fraction of treated patients exhibiting clinical response, there is an urgent need to identify predictive biomarkers. Expression of immune checkpoint genes such as PD-1, PD-L1, and CTL-4 have been reported as predictive biomarkers for tumor immunotherapy response (37, 38). In studying the relationship between migrasome and checkpoint gene expression, we found that migrasome showed a strong correlation with checkpoint gene expression. It suggests that the migrasome may be related to the patient’s response to immunotherapy. We further evaluated ESTIMATE scores for migrasome in a variety of tumors and found that they were higher in patients treated with immunotherapy, implying that they may have a better prognosis. TIDE was further evaluated, and we discovered that high migrasome expression was connected with higher TIDE scores, with higher scores tending to tumor immune escape. Migrasome is also rich in various signaling molecules such as cytokines, growth factors, and chemokines, all of which may play an important role in mediating immune escape from a variety of tumors. In pancreatic cancer cells, migrasome is rich in chemokines such as CXCL5 and cytokines such as TGF-β1, which can be released into the surrounding environment to recruit immune cells and induce their differentiation into immunosuppressive and tumor-promoting phenotypes, further promoting malignant biological functions and immune escape in pancreatic cancer (6).

In this study, we performed a pan-cancer analysis of the migrasome in a variety of cancers and explored the relationship between its aberrant expression and patient survival prognosis. However, our study also has some limitations. First, the number of cancer types included in our study is limited and not very comprehensive, and data on certain specific cancer types are currently unavailable. Second, while data analysis and preliminary experimental verification on migrasome in human cancers has gained some meaningful insights, validation of these findings through animal or cellular experiments would be more beneficial for clinical utility. Thirdly, due to the limitations of the current study, we were unable to demonstrate the presence of migrasome in all tumors studied through images, and this will be demonstrated in subsequent studies of individual tumors.

## Conclusion

In this study, we performed the first pan-cancer analysis of migrasome and demonstrated that migrasomes were associated with prognosis in a variety of tumors. Additionally, migrasome associated with tumor immune escape has potential as an immune checkpoint in cancer therapy. In conclusion, our comprehensive pan-cancer analysis of migrasome shows the characterization of migrasome in a variety of cancer types and provides insights for future migrasome research in clinical tumor prognosis prediction and immunotherapy evaluation.

## Data availability statement

The datasets presented in this study can be found in online repositories. The names of the repository/repositories and accession number(s) can be found below: https://www.ncbi.nlm.nih.gov/, GSE152938.

## Ethics statement

The studies involving human participants were reviewed and approved by Guangxi Medical University Cancer Hospital. The patients/participants provided their written informed consent to participate in this study.

## Author contributions

YQ, JY, CL, JL, ZD, BY, YF, YL, XL, XW, and WL conceived and designed the experiments; YQ, JY, CL, and JL: performed data collection; ZD, BY, YF, YL, XL, XW, and WL analyzed the data; YQ, JY, CL, JL, ZD, BY, YF, YL, XL, XW, and WL helped with the reagents/materials/analysis tools; YQ, JY, CL, JL, ZD, BY, YF, YL, XL, XW, and WL contributed to the writing of the manuscript. All authors reviewed the manuscript. All authors contributed to the article and approved the submitted version.

## Funding

Guangxi Multidisciplinary Collaborative Health Management Talent Mini-Highland (guizutongzi:2019-85).

## Conflict of interest

The authors declare that the research was conducted in the absence of any commercial or financial relationships that could be construed as a potential conflict of interest.

## Publisher’s note

All claims expressed in this article are solely those of the authors and do not necessarily represent those of their affiliated organizations, or those of the publisher, the editors and the reviewers. Any product that may be evaluated in this article, or claim that may be made by its manufacturer, is not guaranteed or endorsed by the publisher.
