# Supplementary material for: Pan-cancer analysis identifies migrasome-related genes as a potential immunotherapeutic target: A bulk omics research and single cell sequencing validation
Source: Front Immunol. 2022 Nov 3;13:994828. doi: 10.3389/fimmu.2022.994828 (PMC9669594; doi:10.3389/fimmu.2022.994828)
Supplement: Supplementary file 12 [file Table_2.docx]

**Supplementary Table 2: Differential expression analysis of MRGs in TCGA tumors and paracancerous.**

|  | TSPAN4 | | NDST1 | | PIGK | | CPQ | | EOGT | | ITGA5 | | ITGB1 | |
| --- | --- | --- | --- | --- | --- | --- | --- | --- | --- | --- | --- | --- | --- | --- |
| Cancer types | P-Values | Direction | P-Values | Direction | P-Values | Direction | P-Values | Direction | P-Values | Direction | P-Values | Direction | P-Values | Direction |
| ACC | 2.22E-4 | Low expression in cancer | 4.43E-4 | Low expression in cancer | 0.08 | - | 0.08 | - | 7.14E-09 | Low expression in cancer | 0.35 | - | 0.12 | - |
| BLCA | 2.85E-05 | Low expression in cancer | 0.05 | - | 0.38 | - | 4.27E-05 | Low expression in cancer | 8.62E-06 | Low expression in cancer | 1.50E-06 | Low expression in cancer | 0.09 | - |
| BRCA | 1.15E-84 | Low expression in cancer | 2.43E-05 | High expression in cancer | 5.01E-49 | High expression in cancer | 2.60E-3 | Low expression in cancer | 1.96E-60 | Low expression in cancer | 6.12E-52 | Low expression in cancer | 1.86E-09 | High expression in cancer |
| CESC | 7.18E-05 | Low expression in cancer | 2.00E-02 | Low expression in cancer | 1.82E-02 | High expression in cancer | 1.93E-05 | Low expression in cancer | 0.05 | - | 6.15E-03 | Low expression in cancer | 0.49 | - |
| CHOL | NA | - | NA | - | NA | - | NA | - | NA | - | NA | - | NA | - |
| COAD | 7.90E-56 | Low expression in cancer | 1.41E-22 | Low expression in cancer | 8.62E-35 | High expression in cancer | 1.02E-38 | Low expression in cancer | 1.44E-26 | Low expression in cancer | 5.39E-57 | Low expression in cancer | 0.96 | - |
| DLBC | 3.22E-28 | High expression in cancer | 2.34E-3 | Low expression in cancer | 1.54E-06 | High expression in cancer | 3.55E-3 | High expression in cancer | 1.63E-20 | Low expression in cancer | 5.60E-4 | Low expression in cancer | 1.40E-09 | High expression in cancer |
| ESCA | 3.27E-54 | Low expression in cancer | 0.09 | - | 1.90E-58 | High expression in cancer | 3.16E-16 | Low expression in cancer | 6.10E-13 | Low expression in cancer | 1.36E-18 | Low expression in cancer | 9.26E-07 | High expression in cancer |
| GBM | 6.71E-39 | High expression in cancer | 1.08E-21 | High expression in cancer | 1.78E-58 | High expression in cancer | 2.92E-52 | High expression in cancer | 1.37E-13 | High expression in cancer | 5.17E-74 | High expression in cancer | 3.338E-68 | High expression in cancer |
| HNSC | 0.16 | - | 0.05 | - | 0.11 | - | 0.71 | - | 0.13 | - | 0.24 | - | 4.71E-02 | High expression in cancer |
| KICH | 1.03E-05 | Low expression in cancer | 8.51E-07 | High expression in cancer | 1.70E-3 | High expression in cancer | 2.22E-10 | High expression in cancer | 4.07E-02 | Low expression in cancer | 2.04E-02 | High expression in cancer | 0.68 | - |
| KIRC | 6.00E-11 | High expression in cancer | 107E-10 | High expression in cancer | 3.22E-13 | High expression in cancer | 1.43E-11 | High expression in cancer | 5.28E-11 | High expression in cancer | 1.74E-15 | High expression in cancer | 6.05E-15 | High expression in cancer |
| KIRP | 1.76E-09 | High expression in cancer | 1.47E-02 | High expression in cancer | 5.38E-08 | High expression in cancer | 9.51E-04 | High expression in cancer | 1.11E-02 | High expression in cancer | 0.05 | - | 2.17E-09 | High expression in cancer |
| LAML | 2.94E-04 | Low expression in cancer | 1.45E-09 | High expression in cancer | 1.28E-04 | High expression in cancer | 1.10E-19 | High expression in cancer | 5.11E-31 | High expression in cancer | 4.18E-10 | High expression in cancer | 3.51E-23 | Low expression in cancer |
| LGG | 2.22E-50 | High expression in cancer | 1.48E-175 | High expression in cancer | 9.72E-100 | High expression in cancer | 0.66 | - | 5.37-11 | Low expression in cancer | 1.23E-56 | High expression in cancer | 8.54E-107 | High expression in cancer |
| LIHC | 1.31E-25 | High expression in cancer | 3.28E-02 | Low expression in cancer | 2.78E-22 | High expression in cancer | 4.16E-50 | High expression in cancer | 2.36E-03 | High expression in cancer | 3.66E-03 | High expression in cancer | 4389E-20 | High expression in cancer |
| LUAD | 3.92E-70 | Low expression in cancer | 1.97E-73 | Low expression in cancer | 5.53E-41 | High expression in cancer | 4.99E-37 | Low expression in cancer | 2.85E-106 | Low expression in cancer | 2.09E-103 | Low expression in cancer | 0..49 | - |
| LUSC | 4.81E-112 | Low expression in cancer | 9.96E-74 | Low expression in cancer | 7.31E-24 | High expression in cancer | 7.49E-72 | Low expression in cancer | 1.11E-106 | Low expression in cancer | 6.13E-90 | Low expression in cancer | 1.75E-08 | Low expression in cancer |
| MESO | NA | - | NA | - | NA | - | NA | - | NA | - | NA | - | NA | - |
| OV | 6.83E-41 | Low expression in cancer | 4.23E-10 | Low expression in cancer | 6.56E-06 | High expression in cancer | 7.81E-42 | Low expression in cancer | 1.26E-42 | Low expression in cancer | 2.90E-23 | Low expression in cancer | 0.77 | - |
| PAAD | 1.49E-45 | High expression in cancer | 7.61E-16 | High expression in cancer | 5.63E-53 | High expression in cancer | 1.28E-48 | High expression in cancer | 1.65E-35 | High expression in cancer | 4.80E-48 | High expression in cancer | 1.00E-53 | High expression in cancer |
| PCPG | 0.63 | - | 0.94 | - | 0.24 | - | 0.10 | - | 0.87 | - | 0.32 | - | 0.73 | - |
| PRAD | 3.02E-42 | Low expression in cancer | 0.59 | - | 4.23E-39 | High expression in cancer | 0.35 | - | 4.09E-49 | Low expression in cancer | 8.21E-47 | Low expression in cancer | 7.60E-3 | High expression in cancer |
| READ | 6.23E-31 | Low expression in cancer | 2.72E-10 | Low expression in cancer | 1.55E-18 | High expression in cancer | 2.55E-17 | Low expression in cancer | 6.66E-12 | Low expression in cancer | 7.23E-29 | Low expression in cancer | 0.77 | - |
| SARC | 0.52 | - | 0.11 | - | 0.24 | - | 0.06 | - | 0.58 | - | 0.61 | - | 0.70 | - |
| SKCM | 1.85E-09 | High expression in cancer | 3.39E-2 | Low expression in cancer | 9.44E-3 | High expression in cancer | 3.38E-12 | High expression in cancer | 1.78E-08 | Low expression in cancer | 1.26E-22 | Low expression in cancer | 0.09 | - |
| STAD | 0.39 | - | 4.22E-22 | High expression in cancer | 3.59E-57 | High expression in cancer | 2.68E-05 | Low expression in cancer | 1.18E-3 | High expression in cancer | 1.33E-2 | Low expression in cancer | 1.12E-30 | High expression in cancer |
| TGCT | 0.61 | - | 0.08 | - | 0.20 | - | 0.39 | - | 5.62E-05 | Low expression in cancer | 1.27E-26 | High expression in cancer | 1.23E-28 | High expression in cancer |
| THCA | 2.37E-19 | Low expression in cancer | 7.17E-11 | Low expression in cancer | 2.35E-26 | High expression in cancer | 4.11E-08 | Low expression in cancer | 3.74E-42 | Low expression in cancer | 1.99E-99 | Low expression in cancer | 1.13E-06 | High expression in cancer |
| THYM | 6.54E-59 | High expression in cancer | 0.88 | - | 5.21E-22 | High expression in cancer | 0.17 | - | 5.93E-58 | Low expression in cancer | 2.22E-11 | Low expression in cancer | 1.03E-31 | High expression in cancer |
| UCEC | 2.64E-35 | Low expression in cancer | 2.58E-35 | Low expression in cancer | 0.29 | - | 4.60E-36 | Low expression in cancer | 1.30E-34 | Low expression in cancer | 9.65E-37 | Low expression in cancer | 3.66E-12 | Low expression in cancer |
| UCS | 2.32E-15 | Low expression in cancer | 4.99E-4 | Low expression in cancer | 0.21 | - | 1.51E-15 | Low expression in cancer | 2.21E-22 | Low expression in cancer | 1.75E-19 | Low expression in cancer | 5.70E-06 | Low expression in cancer |
| UVM | NA | - | NA | - | NA | - | NA | - | NA | - | NA | - | NA | - |
