# Supplementary material for: Pan-cancer analysis identifies migrasome-related genes as a potential immunotherapeutic target: A bulk omics research and single cell sequencing validation
Source: Front Immunol. 2022 Nov 3;13:994828. doi: 10.3389/fimmu.2022.994828 (PMC9669594; doi:10.3389/fimmu.2022.994828)
Supplement: Supplementary file 13 [file Table_3.docx]

**Supplementary Table 3: Correlation of the migrasome-related gene mRNA expression with CNV.**

|  | CNV | | | | | | | | | | | | | |
| --- | --- | --- | --- | --- | --- | --- | --- | --- | --- | --- | --- | --- | --- | --- |
|  | PIGK | | NDST1 | | EOGT | | ITGB1 | | CPQ | | TSPAN4 | | ITGA5 | |
| Cancer types | P-Values | Direction | P-Values | Direction | P-Values | Direction | P-Values | Direction | P-Values | Direction | P-Values | Direction | P-Values | Direction |
| ACC | ≤0.05 | Positive correlation | ≤0.05 | Positive correlation | >0.05 | - | ≤0.05 | Positive correlation | ≤0.05 | Positive correlation | ≤0.05 | Positive correlation | >0.05 | - |
| BLCA | ≤0.05 | Positive correlation | ≤0.05 | Positive correlation | ≤0.05 | Positive correlation | ≤0.05 | Positive correlation | >0.05 | - | ≤0.05 | Positive correlation | >0.05 | - |
| BRCA | ≤0.05 | Positive correlation | ≤0.05 | Positive correlation | ≤0.05 | Positive correlation | ≤0.05 | Positive correlation | ≤0.05 | Positive correlation | ≤0.05 | Positive correlation | >0.05 | - |
| CESC | ≤0.05 | Positive correlation | ≤0.05 | Positive correlation | ≤0.05 | Positive correlation | ≤0.05 | Positive correlation | >0.05 | - | ≤0.05 | Positive correlation | ≤0.05 | Positive correlation |
| CHOL | ≤0.05 | Positive correlation | >0.05 | - | >0.05 | - | >0.05 | - | ≤0.05 | Positive correlation | >0.05 | - | >0.05 | - |
| COAD | ≤0.05 | Positive correlation | ≤0.05 | Positive correlation | ≤0.05 | Positive correlation | ≤0.05 | Positive correlation | >0.05 | - | >0.05 | - | >0.05 | - |
| DLBC | >0.05 | - | >0.05 | - | >0.05 | - | >0.05 | - | >0.05 | - | >0.05 | - | >0.05 | - |
| ESCA | ≤0.05 | Positive correlation | ≤0.05 | Positive correlation | ≤0.05 | Positive correlation | ≤0.05 | Positive correlation | >0.05 | - | ≤0.05 | Positive correlation | >0.05 | - |
| GBM | ≤0.05 | Positive correlation | >0.05 | - | >0.05 | - | ≤0.05 | Positive correlation | >0.05 | - | ≤0.05 | Positive correlation | >0.05 | - |
| HNSC | ≤0.05 | Positive correlation | ≤0.05 | Positive correlation | ≤0.05 | Positive correlation | ≤0.05 | Positive correlation | ≤0.05 | Positive correlation | ≤0.05 | Positive correlation | >0.05 | - |
| KICH | >0.05 | - | ≤0.05 | Positive correlation | >0.05 | - | ≤0.05 | Positive correlation | ≤0.05 | Positive correlation | >0.05 | - | >0.05 | - |
| KIRC | >0.05 | - | ≤0.05 | Positive correlation | ≤0.05 | Positive correlation | >0.05 | - | ≤0.05 | Positive correlation | >0.05 | - | >0.05 | - |
| KIRP | ≤0.05 | Positive correlation | ≤0.05 | Positive correlation | ≤0.05 | Positive correlation | ≤0.05 | Positive correlation | ≤0.05 | Positive correlation | ≤0.05 | Positive correlation | ≤0.05 | Positive correlation |
| LAML | >0.05 | - | >0.05 | - | >0.05 | - | >0.05 | - | >0.05 | - | >0.05 | - | >0.05 | - |
| LGG | ≤0.05 | Positive correlation | >0.05 | - | >0.05 | - | ≤0.05 | Negative correlation | >0.05 | - | ≤0.05 | Positive correlation | >0.05 | - |
| LIHC | ≤0.05 | Positive correlation | ≤0.05 | Positive correlation | ≤0.05 | Positive correlation | ≤0.05 | Positive correlation | ≤0.05 | Positive correlation | ≤0.05 | Positive correlation | ≤0.05 | Positive correlation |
| LUAD | ≤0.05 | Positive correlation | ≤0.05 | Positive correlation | ≤0.05 | Positive correlation | ≤0.05 | Positive correlation | >0.05 | - | ≤0.05 | Positive correlation | ≤0.05 | Positive correlation |
| LUSC | ≤0.05 | Positive correlation | ≤0.05 | Positive correlation | ≤0.05 | Positive correlation | ≤0.05 | Positive correlation | >0.05 | - | ≤0.05 | Positive correlation | >0.05 | - |
| MESO | ≤0.05 | Positive correlation | >0.05 | - | >0.05 | - | >0.05 | - | >0.05 | - | ≤0.05 | Positive correlation | >0.05 | - |
| OV | ≤0.05 | Positive correlation | ≤0.05 | Positive correlation | >0.05 | - | ≤0.05 | Positive correlation | ≤0.05 | Positive correlation | ≤0.05 | Positive correlation | >0.05 | - |
| PAAD | ≤0.05 | Positive correlation | >0.05 | - | ≤0.05 | Positive correlation | >0.05 | - | >0.05 | - | >0.05 | - | >0.05 | - |
| PCPG | ≤0.05 | Positive correlation | >0.05 | - | ≤0.05 | Positive correlation | >0.05 | - | ≤0.05 | Positive correlation | >0.05 | - | >0.05 | - |
| PRAD | ≤0.05 | Positive correlation | ≤0.05 | Positive correlation | >0.05 | - | ≤0.05 | Positive correlation | >0.05 | - | >0.05 | - | >0.05 | - |
| READ | ≤0.05 | Positive correlation | >0.05 | - | >0.05 | - | >0.05 | - | ≤0.05 | Positive correlation | >0.05 | - | >0.05 | - |
| SARC | ≤0.05 | Positive correlation | ≤0.05 | Positive correlation | ≤0.05 | Positive correlation | ≤0.05 | Positive correlation | ≤0.05 | Positive correlation | ≤0.05 | Positive correlation | ≤0.05 | Negative correlation |
| SKCM | ≤0.05 | Positive correlation | ≤0.05 | Positive correlation | ≤0.05 | Positive correlation | ≤0.05 | Positive correlation | >0.05 | - | ≤0.05 | Positive correlation | ≤0.05 | Positive correlation |
| STAD | ≤0.05 | Positive correlation | ≤0.05 | Positive correlation | ≤0.05 | Positive correlation | ≤0.05 | Positive correlation | >0.05 | - | ≤0.05 | Positive correlation | >0.05 | - |
| TGCT | ≤0.05 | Positive correlation | >0.05 | - | >0.05 | - | ≤0.05 | Positive correlation | >0.05 | - | ≤0.05 | Positive correlation | >0.05 | - |
| THCA | >0.05 | - | >0.05 | - | >0.05 | - | >0.05 | - | >0.05 | - | >0.05 | - | >0.05 | - |
| THYM | >0.05 | - | >0.05 | - | >0.05 | - | >0.05 | - | >0.05 | - | ≤0.05 | Positive correlation | >0.05 | - |
| UCEC | >0.05 | - | >0.05 | - | >0.05 | - | ≤0.05 | Positive correlation | ≤0.05 | Positive correlation | >0.05 | - | >0.05 | - |
| UCS | >0.05 | - | ≤0.05 | Positive correlation | ≤0.05 | Positive correlation | ≤0.05 | Positive correlation | ≤0.05 | Positive correlation | ≤0.05 | Positive correlation | >0.05 | - |
| UVM | ≤0.05 | Positive correlation | >0.05 | - | >0.05 | - | >0.05 | - | >0.05 | - | >0.05 | - | ≤0.05 | Positive correlation |
