# Supplementary material for: Pan-cancer analysis identifies migrasome-related genes as a potential immunotherapeutic target: A bulk omics research and single cell sequencing validation
Source: Front Immunol. 2022 Nov 3;13:994828. doi: 10.3389/fimmu.2022.994828 (PMC9669594; doi:10.3389/fimmu.2022.994828)
Supplement: Supplementary file 14 [file Table_4.docx]

**Supplementary Table 4: The methylation levels of migrasome-related genes between tumor and normal samples for each cancer.**

|  | Methylation differences | | | | | | | | | |
| --- | --- | --- | --- | --- | --- | --- | --- | --- | --- | --- |
|  | PIGK | | ITGA5 | | ITGB1 | | TSPAN4 | | NDST1 | |
| Cancer types | P-Values | Direction | P-Values | Direction | P-Values | Direction | P-Values | Direction | P-Values | Direction |
| ACC | NA | - | NA | - | NA | - | NA | - | NA | - |
| BLCA | ≤0.05 | High Methylation | ≤0.05 | High Methylation | ≤0.05 | High Methylation | ≤0.05 | High Methylation | ≤0.05 | High Methylation |
| BRCA | ≤0.05 | High Methylation | ≤0.05 | High Methylation | ≤0.05 | High Methylation | ≤0.05 | High Methylation | ≤0.05 | High Methylation |
| CESC | NA | - | NA | - | NA | - | NA | - | NA | - |
| CHOL | NA | - | NA | - | NA | - | NA | - | NA | - |
| COAD | NA | - | ≤0.05 | High Methylation | NA | - | ≤0.05 | High Methylation | ≤0.05 | High Methylation |
| DLBC | NA | - | NA | - | NA | - | NA | - | NA | - |
| ESCA | NA | - | NA | - | NA | - | ≤0.05 | High Methylation | NA | - |
| GBM | NA | - | NA | - | NA | - | NA | - | NA | - |
| HNSC | ≤0.05 | High Methylation | ≤0.05 | Low Methylation | ≤0.05 | High Methylation | ≤0.05 | High Methylation | ≤0.05 | High Methylation |
| KICH | NA | - | NA | - | NA | - | NA | - | NA | - |
| KIRC | ≤0.05 | High Methylation | ≤0.05 | Low Methylation | ≤0.05 | High Methylation | ≤0.05 | Low Methylation | ≤0.05 | Low Methylation |
| KIRP | ≤0.05 | High Methylation | ≤0.05 | High Methylation | ≤0.05 | High Methylation | ≤0.05 | Low Methylation | ≤0.05 | High Methylation |
| LAML | NA | - | NA | - | NA | - | NA | - | NA | - |
| LGG | NA | - | NA | - | NA | - | NA | - | NA | - |
| LIHC | ≤0.05 | High Methylation | NA | - | ≤0.05 | Low Methylation | ≤0.05 | Low Methylation | NA | - |
| LUAD | NA | - | ≤0.05 | High Methylation | NA | - | ≤0.05 | High Methylation | ≤0.05 | High Methylation |
| LUSC | ≤0.05 | High Methylation | ≤0.05 | High Methylation | NA | - | ≤0.05 | High Methylation | ≤0.05 | High Methylation |
| MESO | NA | - | NA | - | NA | - | NA | - | NA | - |
| OV | NA | - | NA | - | NA | - | NA | - | NA | - |
| PAAD | ≤0.05 | High Methylation | NA | - | NA | - | NA | - | ≤0.05 | High Methylation |
| PCPG | NA | - | NA | - | NA | - | NA | - | NA | - |
| PRAD | ≤0.05 | High Methylation | ≤0.05 | High Methylation | ≤0.05 | Low Methylation | NA | - | ≤0.05 | High Methylation |
| READ | NA | - | NA | - | NA | - | NA | - | NA | - |
| SARC | NA | - | NA | - | NA | - | NA | - | NA | - |
| SKCM | NA | - | NA | - | NA | - | NA | - | NA | - |
| STAD | NA | - | NA | - | NA | - | NA | - | NA | - |
| TGCT | NA | - | NA | - | NA | - | NA | - | NA | - |
| THCA | NA | - | NA | - | ≤0.05 | Low Methylation | NA | - | NA | - |
| THYM | NA | - | NA | - | NA | - | NA | - | NA | - |
| UCEC | ≤0.05 | High Methylation | ≤0.05 | High Methylation | ≤0.05 | High Methylation | ≤0.05 | High Methylation | ≤0.05 | High Methylation |
| UCS | NA | - | NA | - | NA | - | NA | - | NA | - |
| UVM | NA | - | NA | - | NA | - | NA | - | NA | - |
