# Supplementary material for: Pan-cancer analysis identifies migrasome-related genes as a potential immunotherapeutic target: A bulk omics research and single cell sequencing validation
Source: Front Immunol. 2022 Nov 3;13:994828. doi: 10.3389/fimmu.2022.994828 (PMC9669594; doi:10.3389/fimmu.2022.994828)
Supplement: Supplementary file 15 [file Table_5.docx]

**Supplementary Table 5: Correlation of migrasome scores with clinicopathological features.**

|  | Differential expression | | Tumor stage | | OS | | DSS | | PFI | | TIED | | MSI | | TMB | |
| --- | --- | --- | --- | --- | --- | --- | --- | --- | --- | --- | --- | --- | --- | --- | --- | --- |
| Cancer types | P-Values | Direction | P-Values | Direction | P-Values | Direction | P-Values | Direction | P-Values | Direction | P-Values | Direction | P-Values | Direction | P-Values | Direction |
| ACC | NS | - | 0.42 | - | 0.01 | Protective factor | 0.01 | Protective factor | 0.01 | Protective factor | NA | - | 0.02 | Positive correlation | 0.65 | - |
| BLCA | <0.0001 | Low expression in cancer | 1.10E-5 | High expression in stage III-IV | 0 | Risk factor | 0.01 | Risk factor | 0.02 | Risk factor | 6.75E-05 | Positive correlation | 1.17E-10 | Negative correlation | 0.61 | - |
| BRCA | <0.0001 | Low expression in cancer | 0.84 | - | 0.1 | - | 0.04 | Risk factor | 0.01 | Risk factor | 9.32E-09 | Positive correlation | 3.64E-15 | Negative correlation | 1.86E-22 | Negative correlation |
| CESC | <0.01 | Low expression in cancer | NA | - | 0 | Risk factor | 0 | Risk factor | 0.02 | Risk factor | 6.48E-04 | Positive correlation | 0.27 | - | 1.81E-04 | Negative correlation |
| CHOL | NS | - | 0.31 | - | 0.03 | Risk factor | 0.02 | Risk factor | 1 | - | NA | - | 0.34 | - | 0.91 | - |
| COAD | <0.0001 | Low expression in cancer | 0.15 | - | 0.02 | Risk factor | 0.01 | Risk factor | 0 | Risk factor | 0.02 | Positive correlation | 2.00E-06 | Negative correlation | 2.34E-03 | Negative correlation |
| DLBC | <0.01 | Low expression in cancer | NA | - | 0.12 | - | 0.17 | - | 0.02 | Protective factor | NA | - | 0.91 | - | 0.01 | Negative correlation |
| ESCA | <0.0001 | Low expression in cancer | 0.93 | - | 0.3 | - | 0.01 | Risk factor | 0.01 | Protective factor | 0.06 | - | 0.17 | - | 8.95E-03 | Negative correlation |
| GBM | <0.0001 | High expression in cancer | NA | - | 0.01 | Risk factor | 0.02 | Risk factor | 0 | Risk factor | 0.10 | - | 0.89 | - | 0.84 | - |
| HNSC | <0.05 | High expression in cancer | 0.07 | - | 0.03 | Risk factor | 0 | Risk factor | 0.04 | Risk factor | 4.11E-07 | Positive correlation | 1.20E-08 | Negative correlation | 0.10 | - |
| KICH | NS | - | 0.07 | - | 0.1 | - | 0.03 | Risk factor | 0.01 | Risk factor | NA | - | 0.61 | - | 4.92E-02 | Positive correlation |
| KIRC | <0.0001 | High expression in cancer | 0.28 | - | 0 | Protective factor | 0 | Protective factor | 0.09 | - | 2.66E-06 | Positive correlation | 1.22E-11 | Negative correlation | 1.31E-06 | Negative correlation |
| KIRP | NS | - | 0.27 | - | 0.01 | Risk factor | 0.01 | Risk factor | 0.05 | - | 0.11 | - | 3.70E-05 | Negative correlation | 2.98E-05 | Negative correlation |
| LAML | <0.0001 | High expression in cancer | NA | - | 0.26 | - | NA | - | NA | - | 7.39E-06 | Negative correlation | 1.38E-06 | Negative correlation | 5.99E-05 | Negative correlation |
| LGG | <0.05 | High expression in cancer | NA | - | 0 | Risk factor | 0 | Risk factor | 0 | Risk factor | 6.52E-05 | Negative correlation | 0.10 | - | 1.05E-07 | Positive correlation |
| LIHC | <0.0001 | High expression in cancer | 0.87 | - | 0 | Risk factor | 0 | Risk factor | 0.01 | Risk factor | 4.45E-05 | Negative correlation | 0.07 | - | 0.24 | - |
| LUAD | <0.0001 | Low expression in cancer | 0.83 | - | 0.04 | Risk factor | 0.04 | Risk factor | 0.04 | Risk factor | 0.38 | - | 4.56E-12 | Negative correlation | 3.55E-13 | Negative correlation |
| LUSC | <0.0001 | Low expression in cancer | 0.89 | - | 0 | Risk factor | 0.01 | Risk factor | 0.01 | Risk factor | 0.87 | - | 1.00 | - | 0.40 | - |
| MESO | NS | - | 0.99 | - | 0 | Risk factor | 0.01 | Risk factor | 0.01 | Risk factor | NA | - | 0.65 | - | 0.16 | - |
| OV | <0.0001 | Low expression in cancer | 0.21 | - | 0 | Risk factor | 0 | Risk factor | 0.01 | Risk factor | 8.92E-05 | Positive correlation | 0.80 | - | 0.15 | - |
| PAAD | <0.0001 | High expression in cancer | 0.46 | - | 0.04 | Risk factor | 0.03 | Risk factor | 0.13 | - | 3.50E-03 | Positive correlation | 0.39 | - | 1.60E-02 | Negative correlation |
| PCPG | NS | - | NA | - | 0.07 | - | 0.03 | Protective factor | 0.05 | - | NA | - | 2.85E-2 | Negative correlation | 0.48 | - |
| PRAD | <0.0001 | Low expression in cancer | NA | - | 0.04 | Protective factor | 0.04 | Protective factor | 0.05 | - | NA | - | 3.75E-10 | Negative correlation | 2.12E-02 | Negative correlation |
| READ | <0.0001 | Low expression in cancer | 0.79 | - | 0.07 | - | 0.14 | - | 0.09 | - | NA | - | 0.11 | - | 0.31 | - |
| SARC | NS | - | NA | - | 0.02 | Protective factor | 0.01 | Protective factor | 0.03 | Risk factor | 0.13 | - | 2.10E-10 | Negative correlation | 9.22E-09 | Negative correlation |
| SKCM | NS | - | 0.27 | - | 0.21 | - | 0.23 | - | 0.18 | - | 0.31 | - | 7.69E-3 | Negative correlation | 8.49E-17 | Negative correlation |
| STAD | <0.01 | Low expression in cancer | 0.67 | - | 0 | Risk factor | 0 | Risk factor | 0.02 | Risk factor | 2.27E-05 | Positive correlation | 1.65E-08 | Negative correlation | 6.69E-16 | Negative correlation |
| TGCT | <0.0001 | High expression in cancer | 0.06 | - | 1 | - | 1 | - | 0.01 | Risk factor | NA | - | 0.97 | - | 3.22E-02 | Positive correlation |
| THCA | <0.05 | Low expression in cancer | 0.36 | - | 0.02 | Risk factor | 0.05 | - | 0.04 | Protective factor | NA | - | 0.07 | - | 3.34E-03 | Negative correlation |
| THYM | <0.05 | Low expression in cancer | 0.44 | - | 0.08 | - | 1 | - | 0.16 | - | NA | - | 0.82 | - | 0.05 | - |
| UCEC | <0.0001 | Low expression in cancer | 0.08 | - | 0 | Risk factor | 0 | Risk factor | 0.06 | - | 0.71 | - | 9.23E-06 | Negative correlation | 6.51E-03 | Negative correlation |
| UCS | <0.0001 | Low expression in cancer | 0.15 | - | 0.09 | - | 0.05 | - | 0.2 | - | - | - | 0.59 | - | 0.06 | - |
| UVM | NS | - | 0.63 | - | 0 | Risk factor | 0 | Risk factor | 0 | Risk factor | 0.10 | - | 0.06 | - | 5.68E-03 | Negative correlation |
