# Supplementary material for: Pan-cancer analysis identifies migrasome-related genes as a potential immunotherapeutic target: A bulk omics research and single cell sequencing validation
Source: Front Immunol. 2022 Nov 3;13:994828. doi: 10.3389/fimmu.2022.994828 (PMC9669594; doi:10.3389/fimmu.2022.994828)
Supplement: Supplementary file 16 [file Table_6.docx]

Supplementary Table 5: Comparison of migrasome scores in different KIRC tumor microenvironment cells

| Comparison | Difference | P-value | Significance | L-95%CI | H-95%CI |
| --- | --- | --- | --- | --- | --- |
| CD4+ T cells - CD8+ T cells | 0.036 | 0 | *** | 0.014 | 0.058 |
| CD4+ T cells - Endothelial cells | -0.128 | 0 | *** | -0.149 | -0.107 |
| CD4+ T cells - KIRC1 | -0.005 | 0.5361 |  | -0.02 | 0.01 |
| CD4+ T cells - KIRC2 | 0.028 | 0.0024 | ** | 0.007 | 0.049 |
| CD4+ T cells - KIRC3 | -0.077 | 0 | *** | -0.097 | -0.058 |
| CD4+ T cells - Macrophage | -0.006 | 0.7491 |  | -0.023 | 0.012 |
| CD4+ T cells - Mast cells | 0.064 | 0 | *** | 0.041 | 0.086 |
| CD4+ T cells - Monocyte1 | 0.017 | 0.0635 | . | -0.001 | 0.035 |
| CD4+ T cells - Monocyte2 | 0.005 | 0.5563 |  | -0.011 | 0.02 |
| CD4+ T cells - NK cells | 0.023 | 0.0152 | * | 0.003 | 0.043 |
| CD8+ T cells - Endothelial cells | -0.164 | 0 | *** | -0.188 | -0.14 |
| CD8+ T cells - KIRC1 | -0.041 | 0 | *** | -0.063 | -0.018 |
| CD8+ T cells - KIRC2 | -0.008 | 0.3003 |  | -0.023 | 0.007 |
| CD8+ T cells - KIRC3 | -0.113 | 0 | *** | -0.137 | -0.089 |
| CD8+ T cells - Macrophage | -0.042 | 0 | *** | -0.065 | -0.018 |
| CD8+ T cells - Mast cells | 0.028 | 3.00E-04 | *** | 0.013 | 0.043 |
| CD8+ T cells - Monocyte1 | -0.019 | 0.0686 | . | -0.038 | 0.001 |
| CD8+ T cells - Monocyte2 | -0.031 | 4.00E-04 | *** | -0.052 | -0.011 |
| CD8+ T cells - NK cells | -0.013 | 0.2001 |  | -0.031 | 0.005 |
| Endothelial cells - KIRC1 | 0.123 | 0 | *** | 0.104 | 0.143 |
| Endothelial cells - KIRC2 | 0.156 | 0 | *** | 0.132 | 0.18 |
| Endothelial cells - KIRC3 | 0.051 | 0 | *** | 0.036 | 0.066 |
| Endothelial cells - Macrophage | 0.123 | 0 | *** | 0.105 | 0.141 |
| Endothelial cells - Mast cells | 0.192 | 0 | *** | 0.167 | 0.217 |
| Endothelial cells - Monocyte1 | 0.145 | 0 | *** | 0.123 | 0.168 |
| Endothelial cells - Monocyte2 | 0.133 | 0 | *** | 0.111 | 0.155 |
| Endothelial cells - NK cells | 0.151 | 0 | *** | 0.128 | 0.174 |
| KIRC1 - KIRC2 | 0.033 | 3.00E-04 | *** | 0.011 | 0.055 |
| KIRC1 - KIRC3 | -0.072 | 0 | *** | -0.09 | -0.055 |
| KIRC1 - Macrophage | -0.001 | 0.9159 |  | -0.016 | 0.014 |
| KIRC1 - Mast cells | 0.069 | 0 | *** | 0.045 | 0.092 |
| KIRC1 - Monocyte1 | 0.022 | 0.0216 | * | 0.002 | 0.042 |
| KIRC1 - Monocyte2 | 0.009 | 0.4489 |  | -0.009 | 0.027 |
| KIRC1 - NK cells | 0.028 | 0.0029 | ** | 0.007 | 0.048 |
| KIRC2 - KIRC3 | -0.105 | 0 | *** | -0.128 | -0.082 |
| KIRC2 - Macrophage | -0.034 | 2.00E-04 | *** | -0.056 | -0.011 |
| KIRC2 - Mast cells | 0.036 | 0 | *** | 0.018 | 0.054 |
| KIRC2 - Monocyte1 | -0.011 | 0.3352 |  | -0.029 | 0.007 |
| KIRC2 - Monocyte2 | -0.024 | 0.0115 | * | -0.043 | -0.004 |
| KIRC2 - NK cells | -0.005 | 0.498 |  | -0.02 | 0.01 |
| KIRC3 - Macrophage | 0.072 | 0 | *** | 0.057 | 0.087 |
| KIRC3 - Mast cells | 0.141 | 0 | *** | 0.117 | 0.165 |
| KIRC3 - Monocyte1 | 0.094 | 0 | *** | 0.073 | 0.116 |
| KIRC3 - Monocyte2 | 0.082 | 0 | *** | 0.061 | 0.103 |
| KIRC3 - NK cells | 0.1 | 0 | *** | 0.077 | 0.123 |
| Macrophage - Mast cells | 0.069 | 0 | *** | 0.046 | 0.093 |
| Macrophage - Monocyte1 | 0.023 | 0.0247 | * | 0.002 | 0.044 |
| Macrophage - Monocyte2 | 0.01 | 0.5548 |  | -0.01 | 0.03 |
| Macrophage - NK cells | 0.028 | 0.0029 | ** | 0.007 | 0.05 |
| Mast cells - Monocyte1 | -0.047 | 0 | *** | -0.068 | -0.026 |
| Mast cells - Monocyte2 | -0.059 | 0 | *** | -0.081 | -0.038 |
| Mast cells - NK cells | -0.041 | 0 | *** | -0.061 | -0.021 |
| Monocyte1 - Monocyte2 | -0.013 | 0.097 | . | -0.028 | 0.002 |
| Monocyte1 - NK cells | 0.006 | 0.4633 |  | -0.009 | 0.021 |
| Monocyte2 - NK cells | 0.018 | 0.0441 | * | 0 | 0.036 |
